# Segregation of European Corn Borer, *Ostrinia nubilalis*, Aminopeptidase 1, Cadherin, and Bre5-Like Alleles, from a Colony Resistant to *Bacillus thuringiensis* Cry1Ab Toxins, are not Associated with F_2_ Larval Weights when Fed a Diet Containing Cry1Ab

**DOI:** 10.1673/031.008.2101

**Published:** 2008-03-17

**Authors:** Brad S. Coates, Douglas V. Sumerford, Leslie C. Lewis

**Affiliations:** ^1^ USDA-ARS, Corn Insect and Crop Genetics Research Unit, Genetics Laboratory, Iowa State University, Ames, Iowa 50011.; ^2^ Department of Entomology, Iowa State University, Ames, IA, 50011

**Keywords:** *Ostrinia nubilalis*, single nucleotide polymorphism

## Abstract

Protein receptors may be required for activated *Bacillus thuringiensis* Cry toxins (Cry1Ab) to bind midgut epithelium prior to pore formation. Single nucleotide polymorphism markers from two *Ostrinia nubilalis* Hübner (Lepidoptera: Crambidae) midgut peptide receptors, cadherin (OnCad), aminopeptidase N 1 (OnAPN1), and OnBre5 (Onb3GalT5; a β-1,3-galactosyltransferase family 5 member) were used to examine segregation in F_2_ families derived from paired matings of Cry1Ab-resistant females and Cry1Ab-susceptible males. Genotypic frequencies for these markers did not deviate from Mendelian expectations. Analysis of F_2_ larvae indicate the segregation of single nucleotide pores in OnAPN1, OnBre5 (Onb3GalT5), and OnCad marker loci were independent of the segregation of logio weights of larvae feeding on Cry1Ab diet.

## Introduction

*Bacillus thuringiensis* (Bt) Berliner is a gram-positive soil bacterium originally described as an insect pathogen against Lepidoptera, Diptera, and Coleoptera. Insoluble crystalline inclusion bodies of Bt spores contain three domain toxin proteins called Cry toxins. Toxicity occurs by insertion of oligomerized toxin into midgut epithelial membranes, resulting in formation of pore channels causing osmotic imbalance and sepsis (Schnepf et al. 1998). Susceptibility of Lepidoptera to transgenic *B. thuringiensis* crystalline (Cry) toxins has been shown to occur via interaction with midgut receptors. Cry toxins may bind extracellar domains of cadherin (Vadlamudi et al. 1993; Francis & Bulla, 1995), aminopeptidase N (APN; Knight et al. 1994), or alkaline phosphatase receptors (Jurat-Fuentes et al. 2002). Furthermore, carbohydrate modifications to peptide receptors were shown to enhance toxin-receptor interactions (Knowles et al. 1991; Masson et al. 1995) suggesting that glycosylation may be common among midgut receptors (Griffitts et al. 2001). *Caenorhabditis elegans* Bt resistant (*bre*) mutants evaded membrane pore formation when exposed to Cry5B and Cry14A toxins (Maroquin et al. 2000; Griffitts et al. 2001), and a putative β-1,3-galactosyltransferase family 5 member (b3GalT5) gene was correlated with resistance for the mutant bre5 (Griffitts et al. 2001). Bre5 established the theory that glycosylation pathways that modify midgut peptide receptors can be a mechanism of resistance to Cry toxins.

Feeding by larval stage European corn borer, *Ostrinia nubilalis* Hübner (Lepidoptera: Crambidae), causes economic loss via yield decrease to cultivated corn (Mason et al. 1996). Crop injury caused by *O. nubilalis* has been reduced by transgenic maize hybrids expressing Cry1Ab toxins (Koziel et al. 1993). In 2005, 35% of United States corn acreage was planted with commercial hybrids expressing Cry1Ab toxins (USDA-ERS, 2005). If genetic variance for resistance to Cry1Ab were present in wild populations of *O. nubilalis*, high adoption rates of Bt corn may not only result in a high selection pressure for resistance, but also increase potential for *O. nubilalis* populations to respond to the selection. Failure of transgenic crops due to insect resistance has not been observed in the field, but varying levels of resistance levels of resistance were selected for in laboratory colonies (Bolin et al. 1999; Chaufaux et al. 2001; Alves et al., 2006).

The *O. nubilalis* midgut expresses a 220-kDa cadherin-like protein, and 145- and 154-kDa aminopeptidase (APN) isoforms that bind Cry1Ab (Hua et al. 2001). A full-length cadherin cDNA from *O. nubilalis* was shown to have putative N-glycosylation sites (Coates et al. 2005), and was identified as a major midgut receptor (Flanagan et al. 2005). Reduced trypsin transcript T23 levels were associated with *O. nubilalis* KS-SC colony resistance to native toxins present in Dipel® Bt formulations, but did not show decreased susceptibility to truncated Cry1Ab toxins expressed by transgenic maize (Li et al. 2005). Cry toxin resistance has occurred due to mutations in aminopeptidase N 1 in *Spodoptera exigua* (Herrero et al. 2005) and in cadherin in *Heliothis virescens* (Gahan et al. 2001) and *Pectinophora gossypiella* (Morin et al. 2003). Molecular tools for monitoring *O. nubilalis* cadherin (Coates et al. 2005) and serine protease genes (Coates et al. 2006) were developed, and assessed in pedigrees. Herein we report the use of molecular markers for *O. nubilalis* aminopeptidase N 1 (OnAPN1), OnBre5 (Onb3GalT5), and cadherin (OnCad) genes to assess the relationship between segregation of the candidate-gene markers and Cry1Ab-resistance phenotypes within F_2_ progeny originating from resistant female by susceptible male crosses (Cry1Ab^R^ ♀ × Cry1Ab^S^ ♂).

## Materials and Methods

### Pedigrees and measurement of Cry1Ab resistance traits

A field-collected colony of *O. nubilalis* was exposed to laboratory selection for resistance to Cry1Ab since 2003 (> 25 generations) at USDA-ARS, Corn Insects and Crop Genetics Research Unit (CICGRU), Ames, IA. Resistance ratios were measured by comparing dose response of the Cry1Ab-resistant colony (Cry1Ab^R^) and its parental control colony (Cry1Ab^S^) at their respective LD_50_ values. Dose-response studies and Cry1Ab bioassays employed in the current study use the surface overlay method developed by Marçon et al. (1999). In this method, solutions of trypsinized Cry1Ab toxins are applied to surfaces of an artificial diet that absorbs the toxin. Doses of Cry1Ab used in this study are reported in units of surface area (ng cm^-2^), as difficulties in assessing equivalent doses result when direct comparisons are made to volumetic units (cm^-3^) used by Gahan et al. (2005). During the current study, the LD50 value of the Cry1Ab^S^ colony was 8.9 ng cm^-2^, compared to > 23,000 ng cm^-2^ for the Cry1Ab^R^ colony, thus estimating a resistance ratio of > 2,500-fold. Other Cry1Ab resistant *O. nubilalis* colonies are reported to have resistance ratios of 2,000- to 1,300-fold (Alves et al., 2006). Alves et al., (2006) also assessed resistance via the surface overlay method and found LD_50_ values of 640 to 1000 ng cm^-2^. Cry1Ab^R^ larvae can complete development on freeze-dried, whorl-stage corn tissue containing Cry1Ab, and also survive on reproductive stage corn expressing Cry1Ab (Sumerford, personal observation). In comparison, *P. gossypiella* resistant strains AZP-R and APHIS-98R survive on Cry1Ac toxin concentrations > 10 µg g^-1^ (Tabashnik et al, 2004), and *H. virescens* Cry1Ac resistant strains have an LC_50_ of 506 µg ml^-1^ of diet (Gould et al. 1992; Gould et al., 1995). Direct compassion between µg toxin cm^-2^ and µg toxin ml^-1^ of diet is difficult, but at 23 µg cm^-2^, Cry1Ab^R^ may be considered to show high levels of resistance to Cry1Ab toxin.

**Figure 1.**
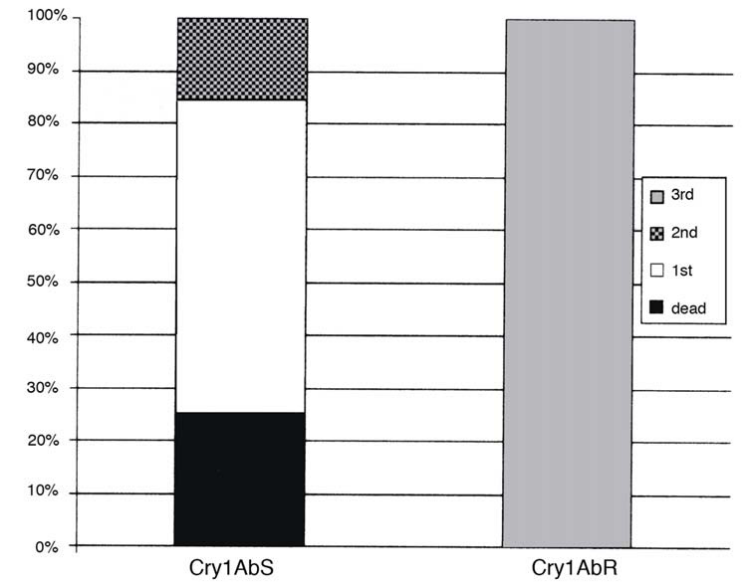
Developmental differences between larvae from Cry1Ab susceptible (Cry1Ab^s^) and Cry1Ab resistant (Cry1Ab^R^) colonies after 7 days feeding on 5.0 ng Cry1Ab toxin cm^-2^.

In order to recover larvae with susceptible phenotypes for DNA extraction, a sublethal bioassay was used as described in other mapping studies involving Bt-resistant colonies (Heckel et al. 1997; Gahan et al. 2005). Larval development of Cry1Ab^R^ individuals is less delayed on sublethal doses of Cry1Ab toxin compared to Cry1Ab^S^. At a dose of 5 and 7.5 ng cm^-2^, Cry1Ab^R^ individuals develop to 3^rd^ instar whereas development of Cry1Ab^S^ larvae is delayed (Figure 1). Development differences, measured as log10-transformed weights (mg), and 5 and 7.5 ng cm^-2^ doses provide a quantitative measure of Cry1Ab resistance and recovery of susceptible individuals for subsequent genetic analysis. Two *O. nubilalis* F1 families (Fam5 and Fam8) were established via paired matings of a female from the Cry1Ab^R^ colony with a Cry1Ab-susceptible male from the CICGRU colony (Cry1Ab^S^). Two matings of full-sib F1 pairs were made from each family for a total of four F_2_ families (Fam 3–14, Fam 3–15, Fam 8–09 and Fam 8–19). F_2_ neonates from each family were fed one of the Cry1Ab-overlay diets (5 and 7.5 ng cm^-2^; 100 – 200 larvae per family), or control overlay (24 larvae) for 7 days, after which larval weights were measured. All larvae were transferred to artificial control diet, reared to adults, and then frozen at -80°C.

### Genotyping assays

Genomic DNA was isolated from parental, F1, and F_2_ adult *O. nubilalis* thorax tissue using DNAeasy isolation kit (Qiagen, www.qiagen.com) according to manufacturer directions. OnAPN1, OnBre5 (Onb3GalT5), and OnCad gene fragments were PCR amplified using 2.5 mM MgCl_2_, 50 µM dNTPs, 2.5 pmol each of primer (Table 1) and, 0.45 U Taq DNA polymerase (Promega, www.promega.com), and 100 ng of DNA template in a 12.5 µl reaction. PTC-100 thermocycler conditions used 95 °C for 2.5 m, followed by 40 cycles of 95 °C for 30 s, 30 s annealing (Table 1), and 72 °C for 1 m. Individual digest reactions included 7.5 µl of *O. nubilalis* Bre5 (Onb3GalT5) or APN1 PCR product, 3.0 µl 10× buffer, 0.1 mg/µl BSA, and 0.25 U of MspI (Onb3Galt5) or RsaI (OnAPN1) in 30 µl, and were incubated at 37 °C for 10 to 16 h. OnCad PCR products (OnCads and OnCad6; Table 1) comprise fragments that amplhy across introns, and both show length variations that were used for allele identification. Entire volumes of all reactions were loaded onto 10 cm 2% agarose gels containing 0.5 µg/ml ethidium bromide, and separated at 100 V for 1 h. Digital images were taken under UV illumination on a BioRad ChemiDoc System (BioRad, Hercules, CA).

**Table 1.**
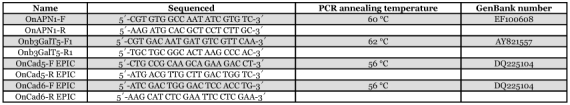
APN1, bre5 (OnB3GaIT5), cadherin primers used in genotyping of *Ostrinia nubilalis* pedigrees.

### Data analysis

F_2_ families were used to determine if OnAPN1, OnBre5 (Onb3GalT5) or OnCad genotypes exhibited Mendelian inheritance, and also to determine if their segregation was related to larval development on sublethal Cry1Ab diets. Replicated goodness-of-fit tests (Sokal and Rohlf 1995) were used to determine if genotype frequencies were inherited in 1:2:1 ratio (Mendelian expectation) at each locus. The replicated goodness-of fit tests produce several G statistics. GH tests whether the frequencies of genotype are homogeneous across all F_2_ families. The pooled-G statistic (Gpooled) tests whether the genotypes pooled across all F_2_ families fit a 1:2:1 Mendelain expectation. Finally, the total G (GTotal) statistic measures whether the data as a whole fit Mendelian expectations. Analyses were performed separately for genotypic data from control and Cry1Ab diets.

**Table 2.**
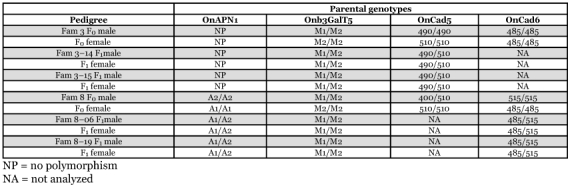
APN1, bre5 (Onb3GalT5), and cadherin alleles observed in pedigrees Fam3–14 and 3–15, and Fam8–09 and Fam8–19. *Ostrinia nubilalis* APN1 *RsaI* PCR-RFLP alleles A1 (310 bp) and A2 (160 and 150 bp); bre5 (Onb3GalT5) *MspI* PCR-RFLP alleles M1 (520, 240, and 127 bp) and M2 (428, 240, 127, and 92 bp). Initial parental cross were between Cry1Ab^R^ femalex Cry1Ab^S^ male.

Marker regressions were used to test the null hypothesis of no relationship among the segregation of resistance phenotypes (log10 weight of larvae feeding on Cry1Ab overlay) with OnAPN1, OnBre5 (Onb3GalT5) and OnCad and genotypes (0, 1, or 2 copies of the allele originating from the Cry1Ab^R^ grandmother; Table 2) within F_2_ families. Regressions were performed using the MIXED procedure of SAS (v. 9.1.3) via restricted-maximum-likelihood methods (Littell et al. 2006). Genotype for each marker was the only fixed effect entered into the model. F_2_ family, block nested within an F_2_ family, and larvae nested with a block (“Residual” error) were considered random sources of variance in their affects on larval logio weight. The relationship between marker genotypes and logio weight was considered significant if *P* ≤ 0.05.

## Results and Discussion

### Mendelian inheritance and larval weights on Cry1Ab diet overlays

Four F_2_ pedigrees (Fam3–14 and 3–15, and Fam8–09 and Fam8–19) were derived from two initial Cry1Ab^R^ ♀ × Cry1Ab^S^ ♂ parental crosses (Fam3 and Fam8). The F0 parents were genotyped using OnAPN1, OnBre5 (Onb3GalT5), and OnCad assays (Table 2). The OnAPN1 RsaI PCR-RFLP marker differentiated parents from Fam8, but no polymorphism was shown between Fam3 parents. All Cry1Ab^R^ female parents were homozygous for the M2 allele at the OnBre5 (Onb3GalT5) locus, and since all Cry1Ab^S^ male parents were heterozygous M1/M2 (sharing the M2 MspI SNP allele observed in the Cry1Ab^R^ colony) all subsequent full-sib crosses were screened in order to select only M1/M2 by M1/M2 for F1 matings. Heterozygous M1/M2 F1 parents in Fam3–14, 3–15, 8–09 and 8–19 allowed determination that the M2 allele was inherited from the Cry1Ab^R^ F_0_ female parent. Two cadherin gene markers, OnCad5 and OnCad6, differentiated parents from Fam3 and Fam8, respectively (Table 2).

Fidelity of allele inheritance is critical in pedigree analysis (Pemberton et al. 1995). Analysis showed that genotypic ratio of F_2_ individuals from Fam3–14, 3–15, 8–09, and 8–19 fed control diet did not deviate from predicted 1:2:1 Mendelian expectations. F_2_ families were homogeneous for observed frequencies of OnAPN1 (GH = 0.08, df = 2, P ≥ 0.9608), OnBre5 (Onb3GalT5; GH = 1.85, df = 6, P ≥ 0.933), and OnCad genotypes (GH = 1.91, df = 6, P ≥ 0.9280). Pooled genotype frequencies also fit 1:2:1 Mendelian expectations for OnAPN1 (GPool = 1.09, df = 2, P ≥ 0.5809), OnBre5 (G_pool_ = 13.90, df = 8, P ≥ 0.084), and OnCad (G_Pool_ = 0.67, df = 2, P ≥ 0.716). In addition, the total G-statistic indicated that all families were homogeneous for Mendelian expectation of genotypic frequencies for OnAPN1 (G_Total_ = 1.017, df = 4, P ≥ 0.8836), OnBre5 (G_Total_ = 5.43, df = 8, P ≥ 0.711), and OnCad (G_Total_ = 2.58, df = 8, P ≥0.9581). Mendelian inheritance of OnAPN1 RsaI PCR-RFLP, OnBre5 (Onb3GalT5) MspI PCR-RFLP, and both cadherin markers among F_2_ progeny fed on control diet suggests their appropriate use in population genetic and mapping experiments.

Similar conclusions were drawn from genotypic data analyzed from F_2_ larvae exposed to sublethal doses of Cry1Ab. The *O. nubilalis* F_2_ families were homogeneous for observed frequencies of OnAPN1 (G_H_ = 2.41, df = 4, P ≥ 0.662), OnBre5 (Onb3GalT5; G_H_ = 7.32, df = 6, P ≥ 0.293), and OnCad genotypes (G_H_ = 1.85, df = 6, P ≥ 0.933). Pooled genotype frequencies also fit 1:2:1 Mendelian expectations for OnAPN1 (G_Pool_ = 3.77, df = 6, P ≥ 0.152), OnBre5 (G_Pool_ = 3.69, df = 2, P ≥ 0.158), and OnCad (G_Pool_ = 0.62, df = 2, P ≥ 0.734). In addition, the total G-statistic indicates that all families were homogeneous for Mendelian expectation of genotypic frequencies for OnAPN1 (G_Total_ = 6.17, df = 6, P ≥ 0.404), OnBre5 (G_Total_ = 11.01, df = 8, P ≥ 0.201), and OnCad (G_Total_ = 5.43, df = 8, P ≥ 0.711). Mendelian ratio of F_2_ progeny after Cry1Ab bioassays indicated that genotypes were randomly present among larvae after bioassay (no genotypes were preferentially culled from survivors), and that subsequent regression analysis would be valid.

### Segregation of marker and resistance phenotype analysis

Regression analysis was used to assess if segregation of OnAPN1 RsaI PCR-RFLP, OnBre5 (Onb3GalT5) MspI PCR-RFLP, or OnCad alleles derived from the Cry1Ab^R^ female parent explain the segregation of logio weights for F_2_ larvae feeding on Cry1Ab diets. Because two F_2_ families were exposed to two Cry1Ab doses (5.0 and 7.5 ng/cm^2^), we examined variance attributed to F_2_ family in the model to determine if logio weights could be combined from both diets into one analysis. Examination of variance components for the random effects in the model supported the pooling of data across doses (percentages of the total variance for family, and replicates within a family were 6.1% and 9.6%, respectively).

Two-way marker regressions were performed to examine the relationship between the segregation of OnBre5 (Onb3GalT5) MspI PCR-RFLP and OnCads alleles for Fam 3–14 and 3–15). The OnBre5 x OnCad5 results suggested that no significant gene interactions were present (F = 0.740, P ≥ 0.3908, df = 1, 190), and no single gene effects were present for OnBre5 (F = 0.42, P ≥ 0.5188, df = 1, 190) or OnCads (F = 0.42, P ≥ 0.5172, df = 1, 190). Similar results were found from a 3-way regression analysis of Fam8–09 and Fam8–19 for OnAPN1 RsaIII PCR-RFLP, OnBre5 (Onb3GalT5) MspI PCR-RFLP, OnCad6 allele for co-segregation with F_2_ Cry1Ab resistance traits (Table 3).

**Table 3.**
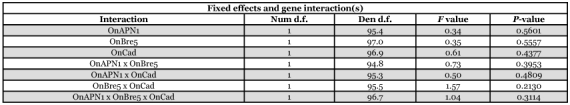
Regression analysis testing for significant 2- and 3-way interaction of genes, OnAPN1, OnBre5 (Onb3GalT5), and OnCad, (0, 1, or 2 copies of the allele derived within the Cry1Ab^R^ colony), with F_2_ larval logio weights from Fam8–09 and 8–19 when fed on diet containing (5.0 and 7.5 ng Cry1Ab/cm^-2^).

Mutation of a single gene product has given rise to Cry1 toxin resistance traits (Gahan et al, 2001, Rajagopal et al. 2002, Herrero et al. 2005). Additionally, it was shown that independent mechanisms might evolve for resistance to different Cry toxins (Jurat-Fuentes et al. 2003), suggesting involvement of multiple midgut receptors (aminopeptidase isoforms, cadherin, and alkaline phosphatase), peptide and lipid modifiers (bre5 homologs), or serine proteases in the spectrum of resistance traits. Bt resistance may have developed by more-than-one independent mechanism in O. nubilalis. Resistance to native Bt toxins in Dipel® formulations was shown to result from decreased expression of an *O. nubilalis* trypsin transcript (T23; Li et al. 2005). In contrast, Siqueira et al. (2004) showed Cry1Ab resistance in the *O. nubilalis* was not associated with decreased serine protease activity, but did show decreased levels of binding at midgut receptors in a Europe-R strain (Siqueira et al., 2006). This evidence suggested midgut receptor binding was a potential point of resistance development in O. nubilalis.

The 3-way analysis of OnAPN1, OnBre5, and OnCad in Fam8–09 and 9–19 tested for epistasis, or gene interaction (Table 3). The F_2_ family was included in the model as a random effect, but showed negligible variance between families (v^2^ = 0.0275) and suggested most variance was contained within families. These analyses also suggest no significant gene interactions were present or that no particular genotype showed a correlation with higher larval log10 weights when reared on sublethal doses of Cry1Ab toxin (P ≥ 0.213). Analysis suggests individually, or in any combination, that OnAPN1, OnBre5, or OnCad might not show significant effect on resistance trait shown by the Cry1Ab^R^ colony.

## Conclusions

This research describes experiments to test correlations between *O. nubilalis* Cry1Ab resistance traits with segregation of alleles at candidate resistance gene loci; aminopeptidase N 1 (APN1), bre5 (Onb3GalT5), and cadherin. Studies with pink bollworm (*P. gossypiella*) indicated three cadherin alleles (r1, r2, and r3) were correlated with Cry1Ac resistance traits (Morin et al. 2003), and the function of cadherins as candidate midgut Bt receptors was shown by a transposon insertion-mediated knockout in *H. virescens* (Gahan et al. 2001). Two *O. nubilalis* Cry1Ab^R^ ♀ × Cry1Ab^S^ ♂ F_2_ pedigrees independently showed a lack relationship between segregation of OnAPN1, Onb3GalT5, or cadherin alleles and factors that affect F_2_ development (larval weight) in Cry1Ab bioassays. Additionally, 2- and 3-way regressions indicated that epistasis (gene interaction) was not involved in resistance traits, suggesting the traits shown by the Cry1Ab^R^ colony might not be polygenic for the genes tested in these experiments.

Additional experiments using other candidate resistance genes such as other aminopeptidases, and alkaline phosphatase, colonies resistant to other Cry toxins, or genomic scans using several genetic markers followed by detection of contributing quantitative trait loci (QTL) will be required to dissect the genetic components of *O. nubilalis* toxin resistance phenotypes.

## Abbreviations

Cry1Ab -: toxins from Bacillus thuringiensis
OnAPN1 -: Ostrinia nubilalis aminopeptidase N isoform 1
OnCad-: Ostrinia nubilalis cadherin
OnBre5 -: Ostrinia nubilalis homolog to Caenorhabditis elegans bre5 gene

## References

[bibr01] Alves AP, Spencer TA, Tabashnik BE, Siegfried BD (2006). Inheritance of resistance to the Cry1Ab *Bacillus thuringiensis* toxin in *Ostrinia nubilalis* (Lepidoptera: Crambidae).. *Journal of Economic Entomology*.

[bibr02] Bolin PC, Hutchinson WD, Andow DA (1999). Long-term selection for resistance to *Bacillus thuringiensis* Cry1Ac endotoxin in a Minnesota population of European corn borer (Lepidoptera: Crambidae).. *Journal of Economic Entomology*.

[bibr03] Chaufaux J, Seguin M, Swanson JJ, Bourguet D, Siegfried BD (2001). Chronic exposure of the European corn borer (Lepidoptera: Crambidae) to Cry1Ab *Bacillus thuringiensis* toxin.. *Journal of Economic Entomology*.

[bibr04] Coates BS, Sumerford DV, Hellmich RL, Lewis LC (2005). Sequence variation in the cadherin gene of *Ostrinia nubilalis*: a tool for field monitoring.. *Insect Biochemistry and Molecular Biology*.

[bibr05] Coates BS, Hellmich RL, Lewis LC (2006). Sequence variation in trypsin- and chymotrypsin-like cDNAs from the midgut of *Ostrinia nubilalis*: Methods for allelic differentiation of candidate *Bacillus thuringiensis* resistance genes. *Insect*.. *Molecular Biology*.

[bibr06] Flanagan RD, Cao-Guo Y, Mathis JP, Meyer TE, Shi X, Siqueira HAA, Siegfried BD (2005). Identification, cloning and expression of a Cry1Ab cadherin receptor from European corn borer, *Ostrinia nubilalis*, (Hübner) (Lepidoptera: Crambidae).. *Insect Biochemistry and Molecular Biology*.

[bibr07] Francis BR, Bulla LA (1997). Further characterization of BT-R_1_, the cadherin-like receptor for Cry1Ab toxin in tobacco hornworm (*Manduca sexta*) midguts.. *Insect Biochemistry and Molecular Biology*.

[bibr08] Gahan LJ, Gould F, Heckel DG (2001). Identification of a gene associated with Bt resistance in *Heliothis virescens*.. *Science*.

[bibr09] Gahan LJ, MacGregor Ma YT, Coble ML, Gould F, Moar WJ, Heckel DG (2005). Genetic basis of resistance to Cry1Ac and Cry2Aa in *Heliothis virescens* (Lepidoptera: Noctuidae).. *Journal of Economic Entomology*.

[bibr10] Gould F, Martínez-Ramírez A, Anderson A, Ferré J, Silva FJ, Moar WJ (1992). Broad-spectrum resistance to *Bacillus thuringiensis* toxin in *Heliothis virescens*.. *Proceedings of the National Academy of Science USA*.

[bibr11] Gould F, Anderson A, Reynolds A, Bumgarner L, Moar W (1995). Selection and genetic analysis of a *Heliothis virescens* (Lepidoptera: Noctuidae) strain with high levels of resistance to *Bacillus thuringiensis* toxins.. *Journal of Economic Entomology*.

[bibr12] Griffitts JS, Whitacre JL, Stevens DE, Aroian RV (2001). Bt toxin resistance from loss of a putative carbohydrate-modifying enzyme.. *Science*.

[bibr13] Herrero S, Gechev T, Bakker PL, Moar WJ, de Maagd RA (2000). *Bacillus thuringiensis* Cry1Ca-resistant *Spodoptera exigua* lacks expression of one of four Aminopeptidase N genes.. *BMC Genomics*.

[bibr14] Hua G, Mason L, Jurat-Fuentes JL, Schwab G, Adang MJ (2001). Binding analysis of *Bacillus thuringiensis* Cryδ -endotoxins using brush border membrane vesicles of *Ostrinia nubilalis*. *Applied and Environmental Microbiology*.

[bibr15] Huang F, Buschman LL, Higgins RA, McGaughey WH (1999). Inheritance of resistance to *Bacillus thuringiensis* toxin (Dipel ES) in the European corn borer.. *Science*.

[bibr16] Jurat-Fuentes JL, Gould FL, Adang MJ (2002). Altered Glycosylation of 63- and 68-kilodalton microvillar proteins in *Heliothis virescens* correlates with reduced Cry1 toxin binding, decreased pore formation, and increased resistance to *Bacillus thuringiensis* Cry1 Toxins.. *Applied and Environmental Microbiology*.

[bibr17] Jurat-Fuentes JL, Gould FL, Adang MJ (2003). Dual resistance to *Bacillus thuringiensis* Cry1Ac and Cry2Aa toxins in *Heliothis virescens* suggests multiple mechanisms of resistance.. *Applied and Environmental Microbiology*.

[bibr18] Knight PJK, Crickmore N, Ellar DJ (1994). The receptor for *Bacillus thuringiensis* Cry1A(c) deltaendotoxin in the brush border membrane of the lepidopteran *Manduca sexta* is aminopeptidase N.. *Molecular Microbiology*.

[bibr19] Knowles BH, Knight PJK, Ellar DJ (1991). N-acetylgalactosamine is part of the receptor in insect gut epithelia that recognizes an insecticidal protein from *Bacillus thuringiensis*.. *Proceedings of the Royal Society of London Biology*.

[bibr20] Koziel MG, Beland GL, Bowman C, Carozzi NB, Crenshaw R, Crossland L, Dawson J, Desai N, Hill M, Kadwell S, Launis K, Lewis K, Maddox D, McPherson K, Meghigi MR, Merlin E, Rhodes R, Warren GW, Wright M, Evola S (1993). Field performance of elite transgenic maize plants expressing an insecticidal protein derived from *Bacillus thuringiensis*.. *BioTechnology*.

[bibr21] Li H, Oppert B, Higgins RA, Huang F, Bushman LL, Gao JR, Zhu KY (2005). Characterization of cDNAs encoding three trypsin-like proteinases and mRNA quantitative analysis in Bt-resistant and -susceptible strains of *Ostrinia nubilalis*.. *Insect Biochemistry and Molecular Biology*.

[bibr22] Littell RA, Milliken GA, Stroup WW, Wolfmger RD, Schabenberger O (2006). *SAS® for mixed models.*.

[bibr23] Marccideljon PCRG, Young LJ, Steffey KL, Siegfried BD (1999). Baseline susceptibility of European corn borer (Lepidoptera: Crambidae) to *Bacillus thuringiensis* toxins.. *Journal of Economic Entomology*.

[bibr24] Marroquin LD, Elyassnia D, Griffitts JS, Feitelson JS, Aroian RV (2000). *Bacillus thuringiensis* (Bt) toxin susceptibility and isolation of resistance mutants in the nematode *Caenorhabditis elegans*.. *Genetics*.

[bibr25] Mason CE, Rice ME, Calvin DD, Van Duyn JW, Showers WB, Hutchison WD, Witkowski JF, Higgins RA, Onstad DW, Dively GP (1996). *European corn borer: Ecology and Management Bull. NC-327*..

[bibr26] Masson L, Lu YJ, Mazza A, Brousseau R, Adang MJ (1995). The CryIA(c) receptor purified from *Manduca sexta* displays multiple specificities.. *Journal of Biological Chemistry*.

[bibr27] Morin S, Biggs RW, Sisterson MS, Shriver L, Ellers-Kirk C, Higginson D, Holley D, Gahan LJ, Heckel DG, Carriere Y, Dennehy TJ, Brown JK, Tabashnik E (2003). Three cadherin alleles associated with resistance to *Bacillus thuringiensis* in pink bollworm.. *Proceeding of the National Academy of Sciences USA*.

[bibr28] Pemberton JM, Slate J, Bancroft DR, Barrett JA (1995). Nonamplifying alleles at microsatellite loci - a caution for parentage and population studies.. *Molecular Ecology*.

[bibr29] Rajagopal R, Sivakumar S, Agrawal N, Malhotra P, Bhatnagar RK (2002). Silencing of midgut aminopeptidase N of *Spodoptera litura* by double-stranded RNA establishes its role as *Bacillus thuringiensis* toxin receptor.. *Journal of Biological Chemistry*.

[bibr30] Schnepf E, Crickmore N, Van Rie J, Lereclus D, Baum J, Feitelson J, Zeigler DR, Dean DH (1988). *Bacillus thuringiensis* and its pesticidal crystal proteins.. *Microbiology and Molecular Biology Reviews*.

[bibr31] Siqueira HAA, Nickerson KW, Moellenbeck D, Siegfried BD (2004). Activity of gut proteinases from Cry1Ab-selected colonies of the European corn borer, *Ostrinia nubilalis* (Lepidoptera: Crambidae).. *Journal of Pest Management Science*.

[bibr32] Siqueira HAA, González-Cabrera J, Ferré J, Flannagan R, Siegfried BD (2006). Analysis of Cry1Ab binding in resistant and susceptible strains of the European corn borer, *Ostrinia nubilalis* (Hübner) (Lepidoptera: Crambidae).. *Applied Environmental Microbiology*.

[bibr33] Sokal RR, Rohlf FJ (1995). *Biometry: the principles and practice of statistics in biological research3*.

[bibr34] Tabashnik BE, Liu YB, Unnithan DC, Carrière Y, Dennehy TJ, Morin S (2004). Shared genetic basis of resistance to Bt toxin Cry1Ac in independent strains of pink bollworm.. *Journal of Economic Entomology*.

[bibr35] Vadlamudi RK, Ji TH, Bulk LA (1993). A specific binding protein from *Manduca sexta* for the insecticidal toxin of *Bacillus thuringiensis* subsp. *Berliner*.. *Journal of Biological Chemistry*.

